# Online monitoring of methane transfer rates unveils nitrogen fixation dynamics in *Methylococcus capsulatus*

**DOI:** 10.1002/bit.28855

**Published:** 2024-10-11

**Authors:** Dominik Engel, Maximilian Hoffmann, Udo Kosfeld, Marcel Mann

**Affiliations:** ^1^ AVT‐Biochemical Engineering RWTH Aachen University Aachen Germany

**Keywords:** gas fermentation, greenhouse gas, methane, methane transfer rate, *Methylococcus capsulatus* (Bath), RAMOS

## Abstract

This study explores methane utilization by the methanotrophic microorganism *Methylococcus capsulatus* (Bath) for biomass production, presenting a promising approach to mitigate methane emissions and foster the development sustainable biomaterials. Traditional screening methods for gas cultivations involve either serum flasks without online monitoring or costly, low‐throughput fermenters. To address these limitations, the Respiration Activity MOnitoring System was augmented with methane sensors for real‐time methane transfer rate (MTR) monitoring in shake flasks. Utilizing online monitoring of the MTR in shake flasks results in enhanced throughput and cost‐effectiveness for cultivating *M. capsulatus*. Simultaneous monitoring of transfer rates for oxygen, methane, and carbon dioxide was conducted in up to eight shake flasks, ensuring the success of the cultivation process. Alterations in methane‐to‐oxygen transfer rate ratios and carbon fixation rates reveal the impact of transfer limitations on microbial growth. Detection of gas transfer limitations, exploration of process parameter influences, and investigations of medium components were enabled by the introduced method. Optimal nitrogen concentrations could be determined to ensure optimal growth. This streamlined approach accelerates the screening process, offering efficient investigations into metabolic effects, limitations, and parameter influences in gas fermentations without the need for elaborate offline sampling, significantly reducing costs and enhanced reproducibility.

## Graphical abstract

Integrating methane sensors into the Respiration Activity Monitoring System (RAMOS) enables real‐time measurement of methane transfer rates in shake flasks. This enhanced method allows for high‐throughput, cost‐effective cultivation of methanotrophic microorganisms while allowing the investigation of gas transfer limitations, process parameters, and medium components.

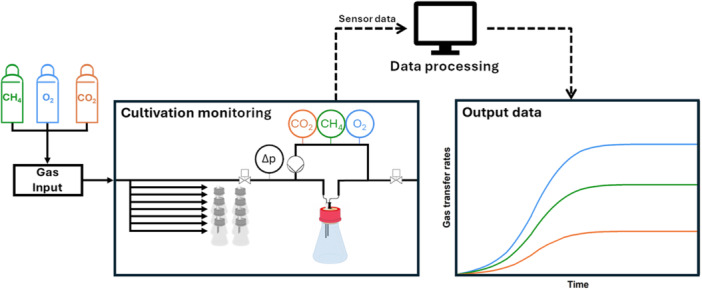

**Abbreviations**
ATEX: ATmosphéres EXplosibles
${\text{CH}}_{4}$
: methane
${\text{CO}}_{2}$
: carbon dioxide
CQ: carbon quotient
CTR: carbon dioxide transfer rate
DI water: deionized water
GGTR: gross gas transfer rate
GHG: greenhouse gas
$H_{2}$
: hydrogen
HPLC: high‐performance liquid chromatography
${\text{KNO}}_{3}$
: potassium nitrate
*M. capsulatus*
: *Methylococcus capsulatus*
MTR: methane transfer rate
${\text{MTR}}_{\text{max}}$
: maximum methane transfer rate
$N_{2}$
: nitrogen
${\text{Na}}_{2}{\text{HPO}}_{4}\cdot 12H_{2}O$
: sodium phosphate dibasic dodecahydrate
$O_{2}$
: oxygen
${\text{OD}}_{600}$
: optical density at 600 nm
OQ: oxidation quotient
OTR: oxygen transfer rate
${\text{OTR}}_{\text{max}}$
: maximum oxygen transfer rate
RAMOS: Respiration Activity MOnitoring System
rpm: Revolutions Per Minute
RQ: respiratory quotient
$\mu_{\text{max}}$
: maximum specific growth rate coefficient

## 1 INTRODUCTION

In the fight against climate change, the most predominate greenhouse gas (GHG) is carbon dioxide (${\text{CO}}_{2}$), followed closely by methane (${\text{CH}}_{4}$), which is less abundant but significantly more potent per mol (Chidambarampadmavathy et al., 2015; Conrad, 2009; Shukla et al., 2022). ${\text{CH}}_{4}$ is 25 to 84 times more potent GHG over 20 years than ${\text{CO}}_{2}$ due to its strong radiative forcing (Allen, 2015; Isaksen et al., 2011; Neubauer, 2014; Pachauri & Mayer, 2015; Solomon et al., 2007). While ${\text{CO}}_{2}$ is the main focus, due to its substantial amounts that is produced yearly, ${\text{CH}}_{4}$ plays a significant factor as well with several initiatives being set in place to reduce its emissions (Nisbet et al., 2020; Shindell et al., 2012). Up to 63% of the atmospheric ${\text{CH}}_{4}$ originates from human activity with 6875 million metric tons being released into the atmosphere every year (Solomon et al., 2007). Excess ${\text{CH}}_{4}$ in the industry is mostly burned for energy production. This further contributes to the accumulation of climate active gas in the atmosphere. Additionally, in Europe, especially in Germany, significant methane production capacity through biogas plants is located, which currently channel methane primarily into energy production. However, with upcoming reductions in government funding through the EEG, there will be a shift towards alternative, nonenergy usage (FNR, 2019; OIES, 2019). For one of those alternative applications, methane should be considered a valuable source of both carbon and energy for methanotrophs.

Methanotrophic microorganisms are capable of metabolizing ${\text{CH}}_{4}$ for diverse purposes, including biomass, methanol, or biopolymer formation, as documented in literature (Foster & Davis, 1966; Gęsicka et al., 2024; Helm et al., 2006; Karthikeyan et al., 2015; Whittenbury et al., 1970). The concurrent reduction of potential GHG emissions and the generation of valuable products, such as animal feed or bioplastics, enhances the attractiveness of these microbial processes for industrial applications. The model organism *Methylococcus capsulatus* (Bath) stands out as the current state‐of‐the‐art for methanotrophic bacteria (Chidambarampadmavathy et al., 2017; Gęsicka et al., 2021). Notably, *M. capsulatus* has been reported to possess the ability to fix atmospheric nitrogen ($N_{2}$) (Chidambarampadmavathy et al., 2017; Murrell & Dalton, 1983). This capability provides a significant advantage over other methanotrophs, allowing for the cultivation of *M. capsulatus* without the need for additional nitrogen sources in the medium. Understanding the impact of varying nitrogen levels and exploring growth dynamics in the absence of nitrogen in the culture broth (e.g., potential reductions in growth or increases in product formation) is crucial for optimizing these processes (Pieja et al., 2012). The combination of screening for diverse microorganisms and optimizing cultivation processes is essential to successfully achieve the outlined objectives. This is of particular importance in gas fermentations where gas transfer limitations are a common issue (Munasinghe & Khanal, 2010; Pauss et al., 1990; Yasin et al., 2015).

However, existing methods for gas fermentations screening and optimization processes often face limitations: they are either too slow and costly when implemented in bioreactors or lack the capability to monitor critical parameters, such as gas transfer rates, in serum flasks. Furthermore, serum flasks, which are not continuously gassed, exhibit a dynamic change in gas composition over time. This varying gas composition poses additional challenges in comparing serum flasks results to large‐scale experiments. Cultivating methanotrophic bacteria poses an additional challenge, primarily attributed to the necessary gas composition utilized for aeration. While oxygen ($O_{2}$) is a common gas for aeration, ${\text{CH}}_{4}$ is not, due to its possible explosive nature in combination with $O_{2}$. Working with ${\text{CH}}_{4}$ requires special safety precautions if both $O_{2}$ and ${\text{CH}}_{4}$ are used in the same process. The required ATmosphéres EXplosibles (ATEX) precautions can be costly and time consuming, hindering the screening of methanotrophs even further.

In this paper, three gases are of importance: $O_{2},{\text{CH}}_{4}$, and ${\text{CO}}_{2}$. While $O_{2}$ and ${\text{CH}}_{4}$ are consumed, ${\text{CO}}_{2}$ is produced during cultivation processes. The changes of the partial pressures of these gases are reflected in the transfer rates and correlate with the metabolic activity of the bacteria. Transfer rates offer vital insights into the processes by providing information about growth rates, gas transfer limitations and metabolic effects over the course of cultivation. With monitored oxygen transfer rate (OTR) and carbon dioxide transfer rate (CTR), the respiratory quotient (RQ) can serve as an indicator for the current consumed carbon source and much more (Anderlei et al., 2004; Finger et al., 2023; Pastoors et al., 2023). In addition to the transfer rates themselves, the ratios between them can also provide essential information. With the additional ${\text{CH}}_{4}$ measurements, additional ratios can be calculated: the oxidation quotient (OQ) and the carbon quotient (CQ). The OQ describes the ratio of needed $O_{2}$ to metabolize the consumed ${\text{CH}}_{4}$ and should give insights into the carbon and energy metabolization of the cultures. Analogous, the CQ is the ratio of consumed and respired carbon in the form of ${\text{CH}}_{4}$ and ${\mathrm{CO}}_{2}$, respectively. CQ can be used to monitor the biomass production and the efficiency of the ${\text{CH}}_{4}$ metabolization.

This work aims to develop a method to monitor ${\text{CH}}_{4}$ transfer rates in shake flasks based on the existing Respiration Activity MOnitoring System (RAMOS) technology. RAMOS is a noninvasive method to monitor the OTR and CTR in shake flasks (Anderlei & Büchs, 2001; Anderlei et al., 2004). The technology has been firmly established for screening in shake flasks for both aerobic cultivations, as well as anaerobic cultivations (Mann et al., 2021; Munch et al., 2020). To further increase the capabilities of the RAMOS technology, methane transfer rate (MTR) monitoring was added to the RAMOS technology to cultivate methanotrophs while monitoring all gases involved in the process. This newly extended device will be referred as the methane RAMOS device. It allows the screening of methanotrophic bacteria in shake flasks, as well as the optimization of their cultivation conditions and process parameters.

In this work, the cultivation of methanotrophs in the RAMOS device as well as the MTR capabilities of the RAMOS technology are evaluated with *M. capsulatus* as a model organism. Different metabolic effects like transfer limitations and growth on atmospheric nitrogen were investigated to show the capabilities of the methane RAMOS technology in observing both biological phenomena, as well as process limitations.

## 2 MATERIAL AND METHODS

### 2.1 Transfer rate monitoring in RAMOS device

All cultivations were conducted in the RAMOS device adapted from Schulte et al. (2018), Munch et al. (2020), and Mann et al. (2021) to monitor gas transfer rates in shake flasks. In addition to the monitoring of OTR and CTR, ${\text{CH}}_{4}$ sensors were added to monitor the MTR. This new device was labeled methane RAMOS. The in‐gas entered the temperature‐controlled shaker and was distributed over capillaries (∅=0.1 mm) to ensure a homogeneous gas distribution to all eight flasks. Each flask was connected to a measurement loop containing a piezo‐membrane pumps mp6‐gas (Bartels Mikrotechnik) and a variety of sensors. The built‐in sensors included $O_{2}$ sensors (Max‐250, Maxtec), infrared ${\text{CO}}_{2}$ sensors (MSH‐P‐CO2, Dynament) and differential pressure sensors type 26PCA (Maxwell Technologies). The ${\text{CO}}_{2}$ sensors have an operating temperature range from $-20^{\circ }C$ to $+50^{\circ }C$ with a measurement range of 0–5 vol% ±0.05 vol% ${\text{CO}}_{2}$. Additionally to the previously described setup, ${\text{CH}}_{4}$ sensors (INIR‐ME100%, Angst+Pfister Sensors and Power AG, n.d) were added to the device. These digital sensors use an infrared light source to measure the concentration of ${\text{CH}}_{4}$ in the gas phase and generate a temperature compensated digital output. According to the manufacturer's datasheets, the sensors have a measurement range of 0–100 vol% ± 0.1 vol% ${\text{CH}}_{4}$ and their performance is verified for a temperature range from −40 to $+75^{\circ }C$. Humidities from 10% to 90% relative humidity were also verified. To determine the interference of humidity, ${\text{CO}}_{2}$ and hydrogen ($H_{2}$) on the ${\text{CH}}_{4}$ sensors, gas compositions between 0–20 vol% ${\text{CO}}_{2}$, 0–80 vol% $H_{2}$, and humidified nitrogen were tested and no significant effect on the measured ${\text{CH}}_{4}$ concentrations could be observed (Figure S4).

**Figure 1:**
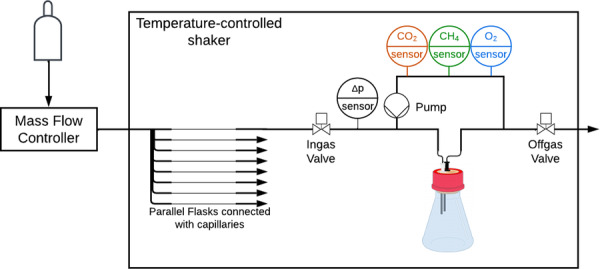
Schematic of the methane Respiration Activity MOnitoring System (RAMOS) device including methane sensors. Gas with a defined composition is introduced into a temperature‐controlled shaker. The in‐gas, controlled by a mass flow controller, is distributed to the eight flasks over capillaries to ensure equal gas distribution between all flasks. Each flask is connected to a measuring loop with $O_{2},{\text{CH}}_{4}$, and ${\text{CO}}_{2}$ sensors. Pressure sensors connected to the loop measure the pressure change in the flasks. During the measurement phase, the valves are closed and the gas is circulated through the measuring loop by piezo‐membrane pumps and the change in gas composition over time is measured.

The gas sensors were combined in a custom 3‐D printed sensor block (DraftGrey) and connected to the measurement loop. A polyjet printer J850 Pro (Stratasys) was used to print the sensor blocks. 3‐D printing enabled rapid prototyping with reduced costs in contrast to manually turned and milled metal blocks (Figure S1). The material itself, as well as the final setup were assessed for gas tightness to prevent gas leakage that could interfere with the measurement. A complete schematic illustration of the whole setup can be found in Figure 1.

The measurement consists of a repeating cycle between an aeration and measurement phase. During the aeration phases, the flasks are continuously aerated to keep a constant gas composition. During the measurement phase, all valves are closed, resulting in a closed system for each flask. The gas is circulated through the measuring loop by the piezo‐membrane pumps and the sensors measure the gas composition over time. The change in gas composition within the headspace of the flasks is used to calculate the transfer rates of the gases. Additionally, the pressure change in the flasks is measured, which is used to calculate the gross gas transfer rate (GGTR). The OTR, CTR, and GGTR were calculated during the measurement phase according to the equations in (Anderlei & Büchs, 2001; Anderlei et al., 2004). In accordance with the other transfer rates, the MTR was calculated according to Equation (1):

(1) $$\text{MTR}=\frac{n_{{\mathrm{CH}}_{4},m}}{V_{L}\cdot t_{m}}=\frac{\Delta p_{{\mathrm{CH}}_{4}}}{\Delta t}\cdot \frac{V_{g}}{R\cdot T\cdot V_{L}},$$

with MTR (mmol/L/h) as the methane transfer rate, $n_{{\text{ch}}_{4},m}$ the moles of ${\text{CH}}_{4}$ (mmol) consumed during the measurement phase, $V_{L}$ (L) the liquid filling volume of the shake flask, $t_{m}$ (h) the duration of the measurement phase, $\Delta p_{{\text{CH}}_{4}}$ (bar) the change of ${\text{CH}}_{4}$ partial pressure in the measurement phase, $V_{g}$ (L) the gas volume in the shake flask, *R* (83.14 bar L/mmol/K) the standard gas constant, and *T* (K) the temperature.

The GGTR is the sum of all consumed gases minus the sum of all produced gases (Equation 2). This can be calculated from the total pressure change in the flasks over the measurement phase. The GGTR equals the sum of the OTR and MTR minus the CTR:

(2) $$\mathrm{GGTR}=\frac{n_{m}}{V_{L}\cdot t_{m}}=\frac{\Delta p}{\Delta t}\cdot \frac{V_{g}}{R\cdot T\cdot V_{L}}=\mathrm{OTR}+\mathrm{MTR}-\mathrm{CTR},$$

with $n_{m}$ the total moles (mmol) consumed during the measurement phase, $V_{L}$ (L) the liquid filling volume of the shake flask, $t_{m}$ (h) the duration of the measurement phase, Δp (bar) the change of total partial pressure in the measurement phase, $V_{g}$ (L) the gas volume in the shake flask, *R* (83.14 bar L/mmol/K) the standard gas constant, and *T* (K) the temperature.

The maximum methane transfer rate (${\text{MTR}}_{\max}$) was calculated from the maximum oxygen transfer rate (${\text{OTR}}_{\max}$) values with a fudge factor of 0.855 to account for the differences in solubility and gas transition coefficients (Equation 3) reported in literature (Cussler, 2009; Ghaz‐Jahanian et al., 2018; Meier et al., 2016; Yu et al., 2006). The following parameters from the experiments were used for the calculations: Osmol: 0.1 Osmol/kg, *n*: 350 revolutions per minute (rpm); $V_{L}$: 10 mL; $d_{0}$: 50 mm; *d*: 85 mm:

(3) $${\text{MTR}}_{\max}={\mathrm{OTR}}_{\max}\cdot 0.855,$$

with the MTR and the already described OTR, the OQ can be calculated analogous to the RQ according to Equation (4):

(4) $$\mathrm{OQ}=\frac{\text{OTR}}{\text{MTR}}.$$

Analogous, the ratio of consumed and exhausted carbon, the CQ, can be calculated with Equation (5):

(5) $$\mathrm{CQ}=\frac{\text{CTR}}{\text{MTR}}.$$

**Figure 2:**
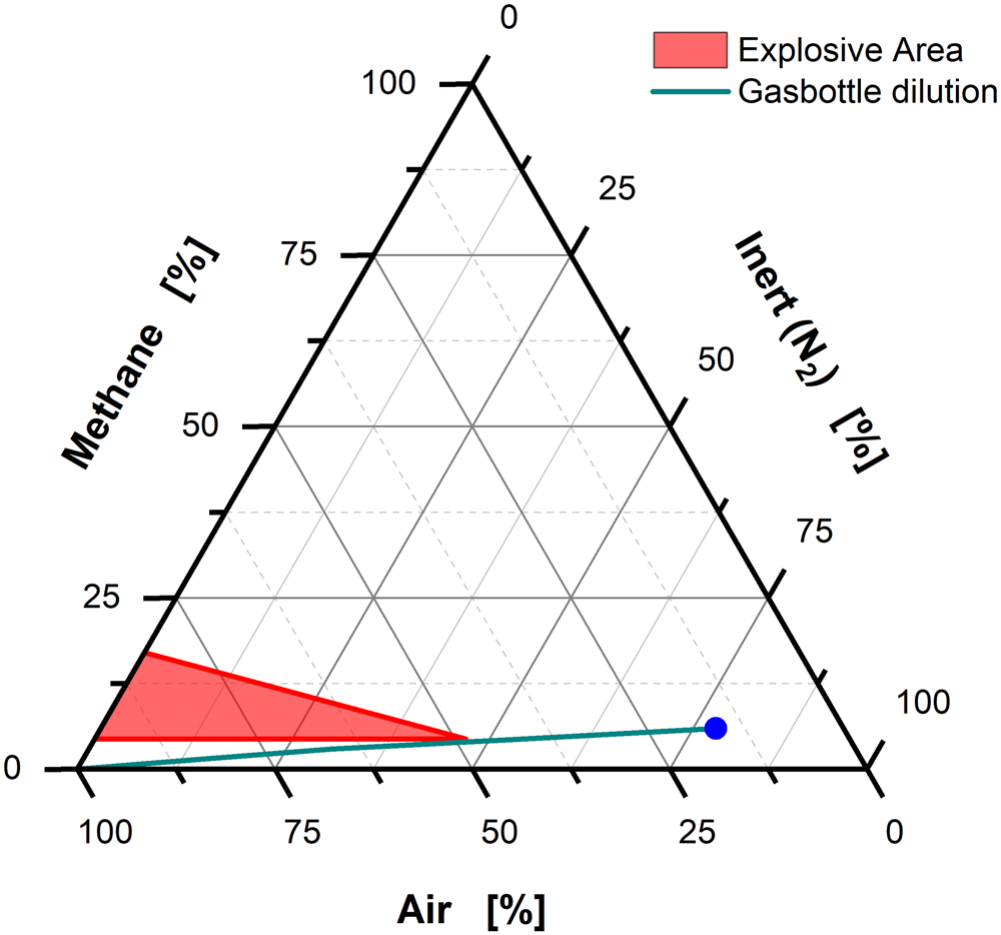
Flammability analysis of ${\text{CH}}_{4}$, air, and inert gas. A ternary diagram displaying the explosive area of ${\text{CH}}_{4}$, air, and inert gas. The red area indicates the explosive area of ${\text{CH}}_{4}$ and air (ATEX). The gas bottle composition is indicated by the blue dot. The green line follows the dilution of the gas if mixed with air. It is to be noted that it never crosses into the explosive area, thus ensuring a safe operation.

Instead of using pressured air or $N_{2}$ to aerate the flasks, a gas mixture of $O_{2},{\text{CH}}_{4}$, and ${\text{CO}}_{2}$ was used. An ATEX consideration was performed before cultivation to ensure safe operation of the device. The chosen gas composition was selected to ensure safe operation of the device by guaranteeing it to stay outside of the explosive area of ${\text{CH}}_{4}$ and air as displayed in Figure 2. The gas was composed of 5.9 vol% ${\text{CH}}_{4}$ and 3.4 vol% $O_{2}$. The blue dot indicates the initial gas composition. In the chart, the red area describes the ATEX state, where $O_{2}$ is available in excess, as well as ${\text{CH}}_{4}$ being present within the lower and upper explosive limit. It is to be noted that the lower explosive limit of ${\text{CH}}_{4}$ (4.4 vol%) is exceeded in the in‐gas (CHEMSAFE, 2014; Prendes‐Gero et al., 2022). In case of a leakage and the gas is diluted with air, the gas composition moves along the green line. Due to the low $O_{2}$ concentration below the limiting oxygen concentration of 9.9 vol% $O_{2}$, by the time that enough $O_{2}$ is present, the ${\text{CH}}_{4}$ concentration is diluted sufficiently to be outside of the explosive area. ${\text{CO}}_{2}$ with a concentration of 1.3 vol% was added to the in‐gas, because it has been reported that an improved growth rate can be observed with an increased ${\text{CO}}_{2}$ concentration in the in‐gas (Henard et al., 2021). Additionally, a co‐utilization of ${\text{CO}}_{2}$ was proposed, which also enhances the growth of methanotrophic organisms (Averesch & Kracke, 2018).

### 2.2 Media composition

All media were prepared using deionized water (DI water). All components were purchased from Carl Roth GmbH & Co, KG, Sigma Aldrich Chemie GmbH, or Merck KGaA. The nitrate mineral salts medium (NMS) was adapted from Petersen et al. (2019). It was prepared as follows: 100 mL/L of nitrate salt stock solution (10 g/L ${\text{MgSO}}_{4}$, 10 g/L ${\text{KNO}}_{3}$, 2 g/L ${\text{CaCl}}_{2}\cdot 2H_{2}O$), 100 mL/L of phosphate buffer solution (2.72 g/L ${\text{KH}}_{2}{\text{PO}}_{4}$, 7.17 g/L ${\text{Na}}_{2}{\text{HPO}}_{4}$), 20 mL/L of 50× iron solution (1 g/L $C_{6}H_{8}O_{7}\cdot {\text{Fe}}_{3}^{3}\cdot {\text{NH}}_{3}$, 2 g/L ${\text{Na}}_{2}{\text{HPO}}_{4}\cdot 12H_{2}O$, 3 mL 37% HCl), 1 mL/L of 1000× trace element solution (0.5 g/L Triplex III, 0.2 g/L ${\text{FeSO}}_{4}\cdot 7H_{2}O$, 0.03 mL $H_{3}{\text{BO}}_{3}$, 0.02 mL ${\text{CoCl}}_{2}\cdot 6H_{2}O$, 0.01 mL ${\text{ZnSO}}_{4}\cdot 7H_{2}O$, 0.003 mL ${\text{MnCl}}_{2}\cdot 4H_{2}O$, 0.003 mL ${\text{Na}}_{2}{\text{MoO}}_{4}\cdot 2H_{2}O$, 0.002 mL ${\text{NiCl}}_{2}\cdot 6H_{2}O$, 2.5 mL ${\text{CuSO}}_{4}\cdot 5H_{2}O$), 17.048 g/L ${\text{CuCl}}_{2}\cdot 2H_{2}O$, 209.27 g/L 3‐(*N*‐morpholino)propanesulfonic acid (MOPS), were diluted with DI water. The pH value was adjusted to pH 6.8 using 1 M KOH. The gas composition for the cultivations was as follows: 5.9 vol% ${\text{CH}}_{4}$, 3.4 vol% $O_{2}$, 1.3 vol% ${\text{CO}}_{2}$, 89.4 vol% $N_{2}$ (Westfalen AG).

### 2.3 Microorganisms

*Methylococcus capsulatus* (Bath) was received from Belgian Coordinated Collections of Microorganisms, 2021. Cryo cultures were prepared from cultures grown on NMS in the RAMOS device over 24 h gassed with the above‐described gas composition. The cells were concentrated by centrifugation and were used to set the optical density at 600 nm (${\text{OD}}_{600}$) of the cryo cultures to 1.25. The strains were stored at $-80^{\circ }C$ with 10 vol% dimethyl sulfoxide in NMS.

### 2.4 Precultures

The precultures were performed in the RAMOS device described in Section 2.1. The medium was inoculated with 80 μL/mL from a cryo culture that had been stored at $-80^{\circ }C$. All precultures were conducted in 250 mL RAMOS flasks with a filling volume of 10 mL with NMS described in Section 2.2. A shaking frequency of 350 rpm and a temperature of $37^{\circ }C$ were used. The precultures were cultivated for 20–30 h and stopped at the late exponential growth phase indicated by an OTR of 12–14 mmol/L/h. Precultures were used to inoculate the main culture with an initial ${\text{OD}}_{600}$ of 0.1.

### 2.5 Offline measurement

The ${\text{OD}}_{600}$ was measured at a wavelength of 600 nm using a Genesys20 photometer (Thermo Fisher Scientific). To determine the cell dry weight, the culture broth was centrifuged for 5 min at 14,000 rpm (Sigma1–15, Sigma) in preweighted 2 mL tubes. The supernatant was discarded or used for subsequent high‐performance liquid chromatography (HPLC) measurements. The remaining cell pellet was dried for at least 24 h at $80^{\circ }C$ and subsequently weighed. For the HPLC measurements, the supernatant was stored at $-20^{\circ }C$. An Ultimate 3000 HPLC system (Thermo Fisher Scientific Inc.) was used. The separating column was equipped with an Organic Acid Resin (300×7.8 mm, Phenomenex Ltd. Deutschland, Aschaffenburg, Germany), and an ERC RefractoMax 520 detector (IDEX Health & Science LLC). The flow rate of the mobile phase (5 mM $H_{2}{\text{SO}}_{4}$) was set to 0.8 mL/min with a column temperature of $60^{\circ }C$. Standards with concentrations between 1.0 and 10.0 g/L were used for all measurements. For the osmolality measurements, an Osmomat 3000 (Gonotec, Logan, USA) was used. Calibration standards of 0.1 and 0.3 Osmol/kg were used for the measurements. For each measurement, 50 μL of supernatant was used.

## 3 RESULTS AND DISCUSSION

### 3.1 Cultivation of *M. capsulatus* in RAMOS device

**Figure 3:**
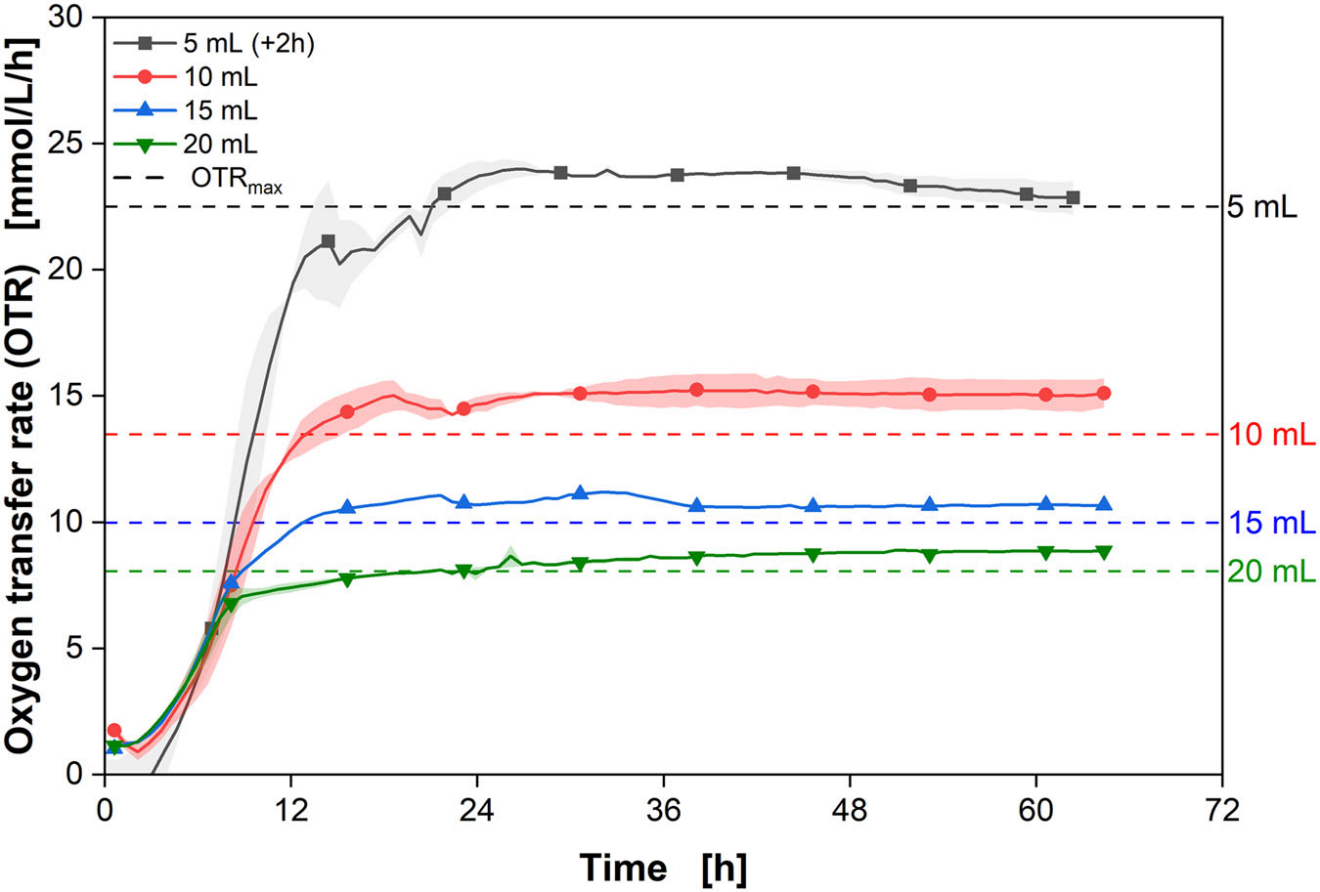
Cultivation of *Methylococcus capsulatus* in Respiration Activity MOnitoring System (RAMOS) device. Oxygen transfer rate (OTR) for different filling volumes of *M. capsulatus* in RAMOS device with ${\text{CH}}_{4}$ continuously supplied as substrate. The theoretical ${\text{OTR}}_{\max}$ values for each filling volume are displayed as dotted lines. The courses of the cultivations with 5 mL filling volume were shifted by 2 h to be aligned with the other courses. The difference in lag time is due to varying initial ${\text{OD}}_{600}$s of the cultures. The experiments were conducted in duplicates, with the average values represented as a line and the min/max values illustrated as error shadows. It is important to note that the filling volume of 15 mL was not monitored in duplicates due to a malfunctioning $O_{2}$ sensor. Cultivation conditions: NMS medium, $c_{\text{buffer}}=30$ mM MOPS), $T=37^{\circ }C$, *n* = 350 rpm, $d_{0}=50$ mm, $V_{L}=5–20$ mL, initial OD = 0.1, initial pH = 6.8, *N* = 1–2. Only every 20th data point over time is indicated by the corresponding symbol; $c_{\text{in‐gas}}=5.9$ vol% ${\text{CH}}_{4}$, 3.4 vol% $O_{2}$, 1.3 vol% ${\text{CO}}_{2}$, 89.4 vol% $N_{2}$.

*M. capsulatus* was cultivated in NMS using the RAMOS device described above with OTR monitor capabilities. This initial approach was crucial for validating both the compatibility of the device with methanotrophic microorganisms and its suitability for the specific gas composition used in the experiments. Similar to the experimental approach outlined by Munch et al. (2020) and Mann et al. (2021), the energy and carbon source, ${\text{CH}}_{4}$, is continuously supplied through the gas phase. Accordingly, the process is a fed‐batch cultivation in respect to the carbon in form of ${\text{CH}}_{4}$. Furthermore, varying filling volumes ranging from 5 to 20 mL were used to investigate the impact of the filling volume on transfer rates. The results are displayed in Figure 3.

All cultures show initial exponential growth with a growth rate of $0.36\;h^{-1}$. This aligns perfectly with the measured maximum specific growth rate coefficient ($\mu_{\max}$) by Zhivotchenko et al. (1995). After 10 h for the high filling volumes to 13 h for the lower filling volumes, the growth of the cultures starts to decelerate. After 19 h, OTR plateaus form at around 23, 14, 11 and 8 mmol/L/h for the filling volumes 5, 10, 15, and 20 mL, respectively. The monitored values of the OTR plateaus decrease with increasing filling volumes. This indicates a gas transfer limitation, as the gas transfer from the gas phase to the liquid phase is limited, preventing higher transfer rates (Anderlei et al., 2004). Additionally, the ${\text{OTR}}_{\max}$ values correlate with the maximum transfer capacity for the tested cultivation conditions (dotted lines) with slight offsets towards lower values.

The correlation from Meier et al. (2016) has been shown to accurately estimate the ${\text{OTR}}_{\max}$ across a wide range of process parameters in shake flasks using air as the in‐gas. For the tested parameters—shaking diameter (1.25–10 cm), flask diameter (51–131 mm), filling volume (2–160 mL), and shaking frequency (100–450 rpm)—the correlation consistently predicts the ${\text{OTR}}_{\max}$ within a range of ±5 mmol/L/h. In this study, the measured ${\text{OTR}}_{\max}$ values were only 1–2 mmol/L/h lower than the predicted values despite a significant reduction in the in‐gas oxygen concentration from 20.9 to 3.4 vol%. This demonstrates that the correlation performs well even under altered gas conditions, with the measured values aligning closely with the predicted range. These results further confirm the validity of the methane RAMOS device for the cultivation of *M. capsulatus* using the given gas composition.

Unlike previous studies that reported sharp turns in $O_{2}$ transfer limitations with air, our study observed a plateau that gradually approaches the ${\text{OTR}}_{\max}$ (Anderlei et al., 2004; Stöckmann et al., 2003). This difference can be attributed to the low $O_{2}$ concentration in our study, which results in a dynamic change in the $O_{2}$ gradient between the gas and liquid phases throughout the measurement phase. During this phase, the $O_{2}$ concentration in the headspace slightly decreases due to consumption by the culture. While such changes are usually negligible in aerobic cultivations, they become more significant in gas fermentation scenarios where the $O_{2}$ content is low. In our case, the $O_{2}$ content is only 3.4 vol% $O_{2}$ and decreases up to 10% during the measurement phase, a stark contrast to aerobic cultivations that have 20.95 vol% $O_{2}$ (Figure S2). An additional effect is the dilution of the $O_{2}$ content with the produced ${\text{CO}}_{2}$. During the exponential growth phase, the $O_{2}$ content in the headspace reduces notably (Figure S3). These two effects seem to influence the $O_{2}$ gradient between the gas and liquid phases, resulting in the gradual formation of a plateau instead of a sharp turn.

This experiment shows that the RAMOS device is applicable for the cultivation of *M. capsulatus*. The different transfer rates can be monitored successfully and expected values are obtained. Moreover, the correlation from Meier et al. (2016) for air (20.95 % $O_{2}$) seems to be applicable for different $O_{2}$ concentrations (here 3.4 vol% $O_{2}$) in the in‐gas as well.

### 3.2 Methane transfer rate monitoring in shake flasks

**Figure 4:**
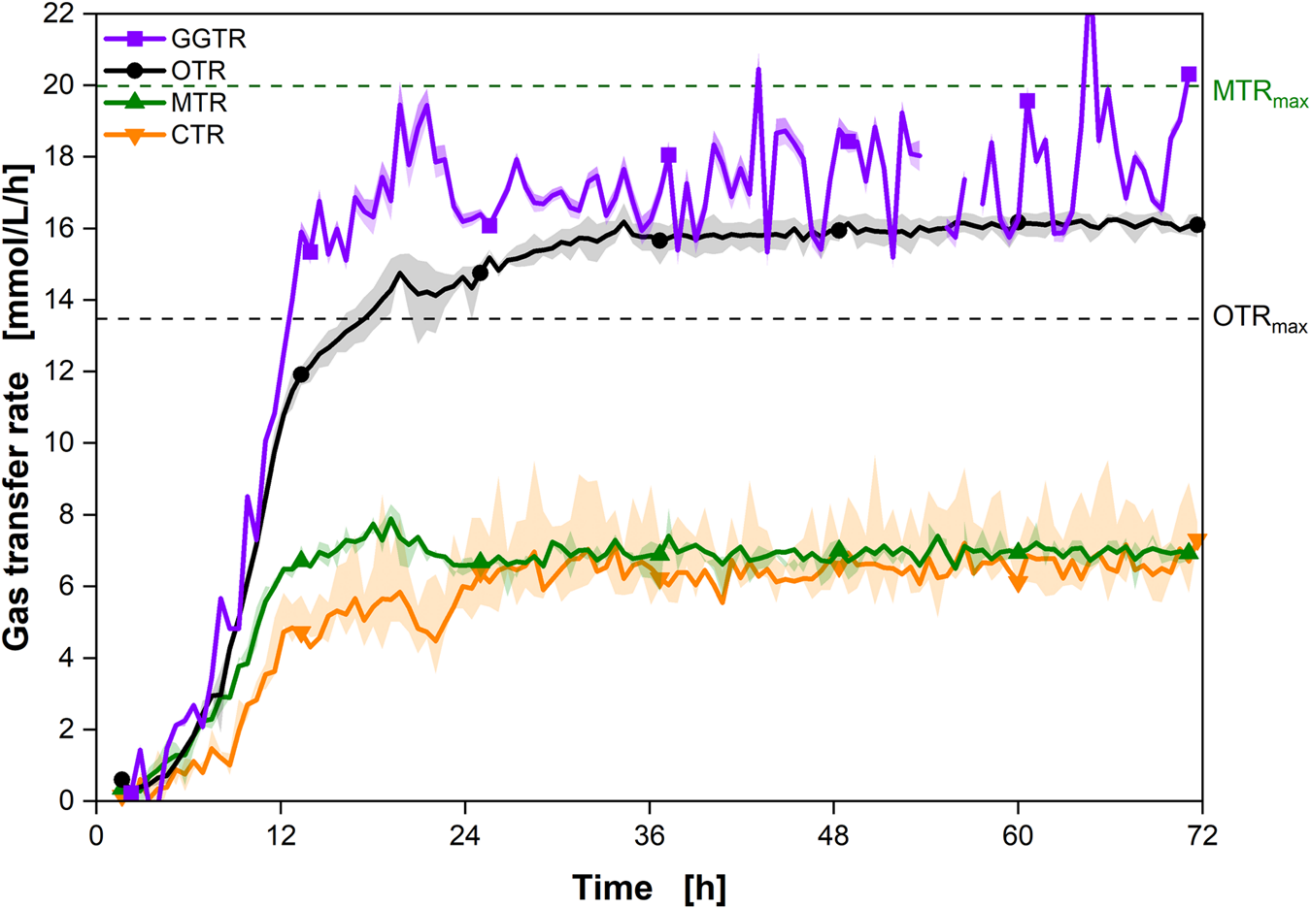
Real‐time monitoring of gas transfer rates, including methane, of *Methylococcus capsulatus* in shake flasks. Online data of the cultivation of *M. capsulatus*, including oxygen transfer rate (OTR), carbon dioxide transfer rate (CTR), methane transfer rate (MTR), and gross gas transfer rate (GGTR). The experiments were conducted in duplicates, with the average values represented as a line and the min/max values illustrated as error shadows. Cultivation conditions: NMS medium, $c_{\text{buffer}}=30$ mM MOPS, $T=37^{\circ }C$, *n* = 350 rpm, $d_{0}=50$ mm, $V_{L}=10$ mL, initial OD = 0.1, initial pH = 6.8, *N* = 3. Only every 20th data point over time is indicated by the corresponding symbol; $c_{\text{in‐gas}}=5.9$ vol% ${\text{CH}}_{4}$, 3.4 vol% $O_{2}$, 1.3 vol% ${\text{CO}}_{2}$, 89.4 vol% $N_{2}$.

After demonstrating the feasibility of the RAMOS device for *M. capsulatus* cultivation, the methane RAMOS described in Section 2.1 was used to cultivate *M. capsulatus* as illustrated in Figure 1. A filling volume of 10 mL was chosen for subsequent cultivations. The monitored transfer rates (OTR, CTR, MTR, and GGTR) are displayed in Figure 4, encompassing all gases being consumed or produced during the experiment. *M. capsulatus* was cultivated in NMS for 72 h to observe the growth and different transfer rates of the culture. The data shown is an average of two biological replicates.

All curves follow a similar pattern to the cultivation in Figure 3, with an initial exponential growth phase. After 12 h, the growth starts to decelerate. After 19 h, a plateau forms at around (15.5±0.33) mmol/L/h for OTR, (7±0.52) mmol/L/h for MTR, and (6.5±1.1) mmol/L/h for CTR. The MTR and CTR align after 26 h. The CTR shows a higher noise signal than the OTR and MTR signals, attributed to one ${\text{CO}}_{2}$ sensor measuring slightly reduced ${\text{CO}}_{2}$ levels. Recalibrating the sensor for subsequent experiments alleviated this discrepancy. While the GGTR is noisier due to the sensitive pressure sensor, a plateau at (17.4±1.38) mmol/L/h can be observed during the limitation.

The plateau in the OTR indicates a gas transfer limitation, as seen in Figure 3. The reached OTR values align with the theoretical ${\text{OTR}}_{\max}$ calculated after Meier et al. (2016), with the observed offset of around 2 mmol/L/h. In contrast, the ${\text{MTR}}_{\max}$ calculated with equation (3) is significantly higher than the observed values, leading to the conclusion that ${\text{CH}}_{4}$ was not limiting during the cultivation. The observed courses strongly suggest the presence of $O_{2}$ limitations. Notably, the transfer rates are intricately linked to the consumption of both $O_{2}$ and ${\text{CH}}_{4}$, with these gases being co‐consumed. Consequently, there exists a restriction on the amount of ${\text{CH}}_{4}$ that can be metabolized, manifesting as observed plateaus. These plateaus represent an additional constraint, resulting in carbon limitation. The final pH of the cultivation was 7.2, which lies well within the pH optimum of the organism (Geymonat et al., 2011; Whittenbury et al., 1970). The increase in pH is either due to the consumption of substrates (e.g., ${\text{KNO}}_{3}$) from the medium or the production of unknown metabolites. Over the course of the cultivation, a total of (436±1.1) mmol/L ${\text{CH}}_{4}$ and (950±23) mmol/L $O_{2}$ were consumed during the cultivation. (385±57) mmol/L ${\text{CO}}_{2}$ were produced. The theoretical ratios of the gases produced and consumed can be derived from the stoichiometric biomass equation described by Villadsen et al. (2011) in equation (6). Similar values were also previously obtained by Sheehan and Johnson (1971) and Petersen et al. (2019).

(6) $$1.45\;O_{2}+{\mathrm{CH}}_{4}=0.52X+0.48{\mathrm{CO}}_{2},$$

$O_{2}$ being oxygen, ${\text{CH}}_{4}$ being methane, X being biomass, and ${\text{CO}}_{2}$ being carbon dioxide. In contrast to the biomass formation, pure combustion of ${\text{CH}}_{4}$ with $O_{2}$ is displayed in Equation (7) and would result in an OQ of 2.0.

(7) $${\mathrm{CH}}_{4}+2\;O_{2}=CO_{2}+2\;H_{2}O.$$

The measured values for consumed $O_{2}$ and ${\text{CH}}_{4}$ and the produced ${\text{CO}}_{2}$ do not fit the expected growth ratios of 1.45 mol/L ${\text{CH}}_{4}$ per 1 mol/L $O_{2}$ and 0.48 mol/L ${\text{CO}}_{2}$ per 1 mmol/L ${\text{CH}}_{4}$ (Equation 6) also reported in literature (Geymonat et al., 2011; Whittenbury et al., 1970). Only for the beginning of the cultivation, the ratios are in line with the theoretical values. These ratios seem to apply only for unlimited growth and shift during transfer limited conditions. The subsequent section provides a detailed analysis of the transfer rates and their respective ratios. This section shows that the methane RAMOS device can monitor all relevant transfer rates of *M. capsulatus* in shake flasks and gas transfer limitations and different transfer rate ratios can be detected.

### 3.3 Molar gas balances

**Figure 5:**
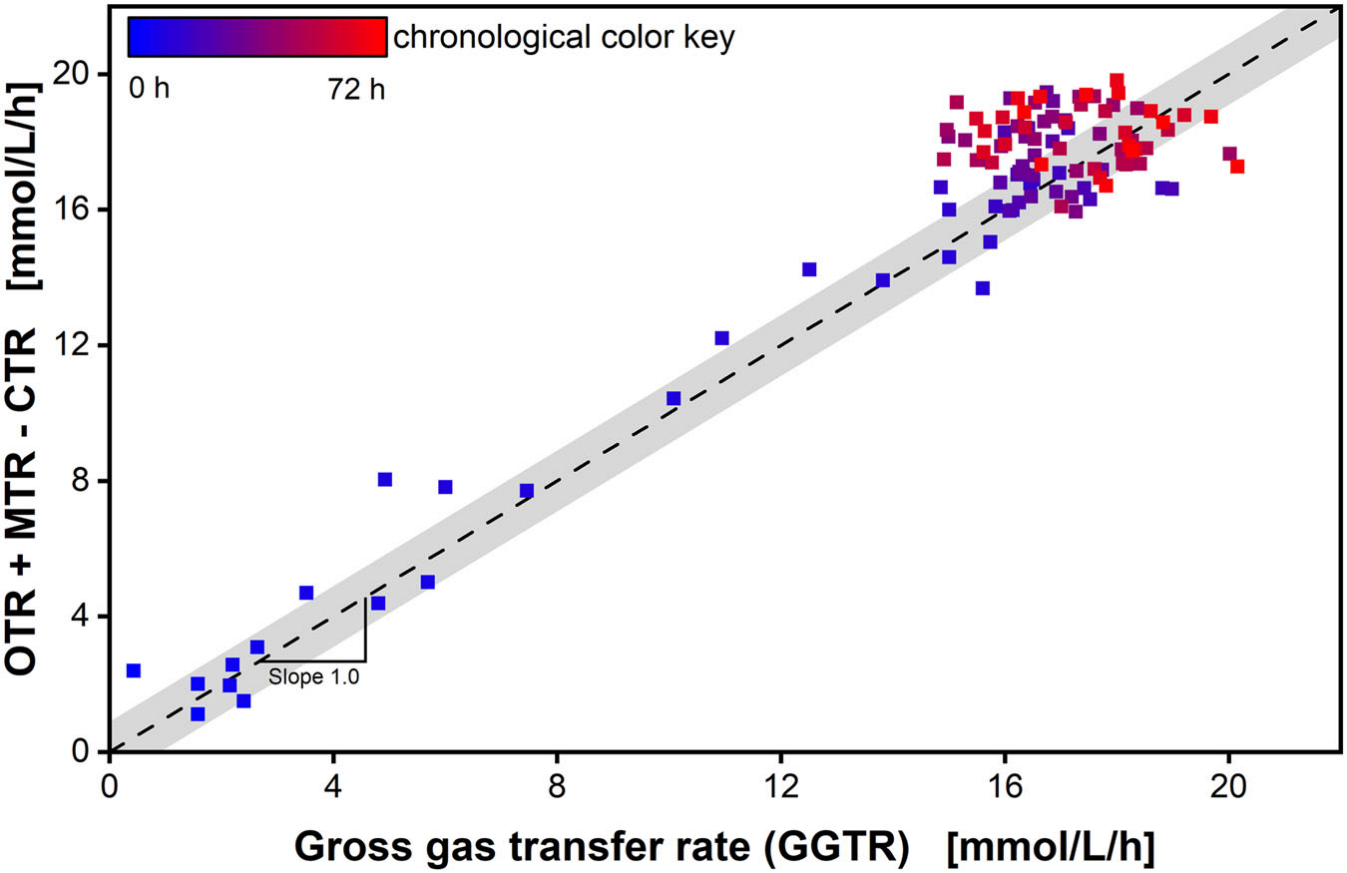
Parity plot of closed gas balances of the cultivation in Figure 4. The combined monitored transfer rates (Equation 2) over the gross gas transfer rate. The color shift from blue to red describes the temporal progression of cultivation time of the cultivation (0–72 h). The bisector, indicated as black dashed line, displays the closed gas balance with a slope of 1. An *R* ^2^ of 0.91 is evident. The standard deviation of the data points with 0.6 mmol/L/h around the bisector indicated as a grey shadow.

Closing the molar gas balance is a good indicator for the validation, as well as the accuracy of the transfer rate monitoring. Methanotrophic microorganisms consume $O_{2}$ and ${\text{CH}}_{4}$ and produce ${\text{CO}}_{2}$ as shown in Equation (6). When considering all gases, the summation of consumed $O_{2}$ and ${\text{CH}}_{4}$, minus the produced ${\text{CO}}_{2}$, should precisely match the total gas produced, as outlined in Equation (2). This equilibrium should manifest as a bisector characterized by a slope of 1. The comparison of the combined gases over the GGTR is displayed in Figure 5. After starting the experiments, the flasks are still filled with air and it takes some time (~1 h) for an equilibrium to be reached within the headspace. This leads to inaccurate measurements for the first data points and thus are ignored for the parity plot analysis.

The data points are distributed around the bisector with a slope of 1, indicating a closed molar gas balance. This clustering indicates a robust alignment of the observed data with the expected trend. A mean of 0.89 mmol/L/h and a deviation of <0.9 mmol/L/h between the theoretical and the measured values can be measured. The *R* ^2^ of 0.91 shows a strong correlation between the monitored and the calculated values. All gas transfer rates are well within acceptable margins, demonstrating precision even in the presence of inherent noise from the pressure sensor. This proofs a closed molar gas balance and a high accuracy of the transfer rate monitoring. These outcomes collectively affirm the robustness and precision of the experimental approach, reinforcing the credibility of the obtained results and underscoring the reliability of the established methodology.

**Figure 6:**
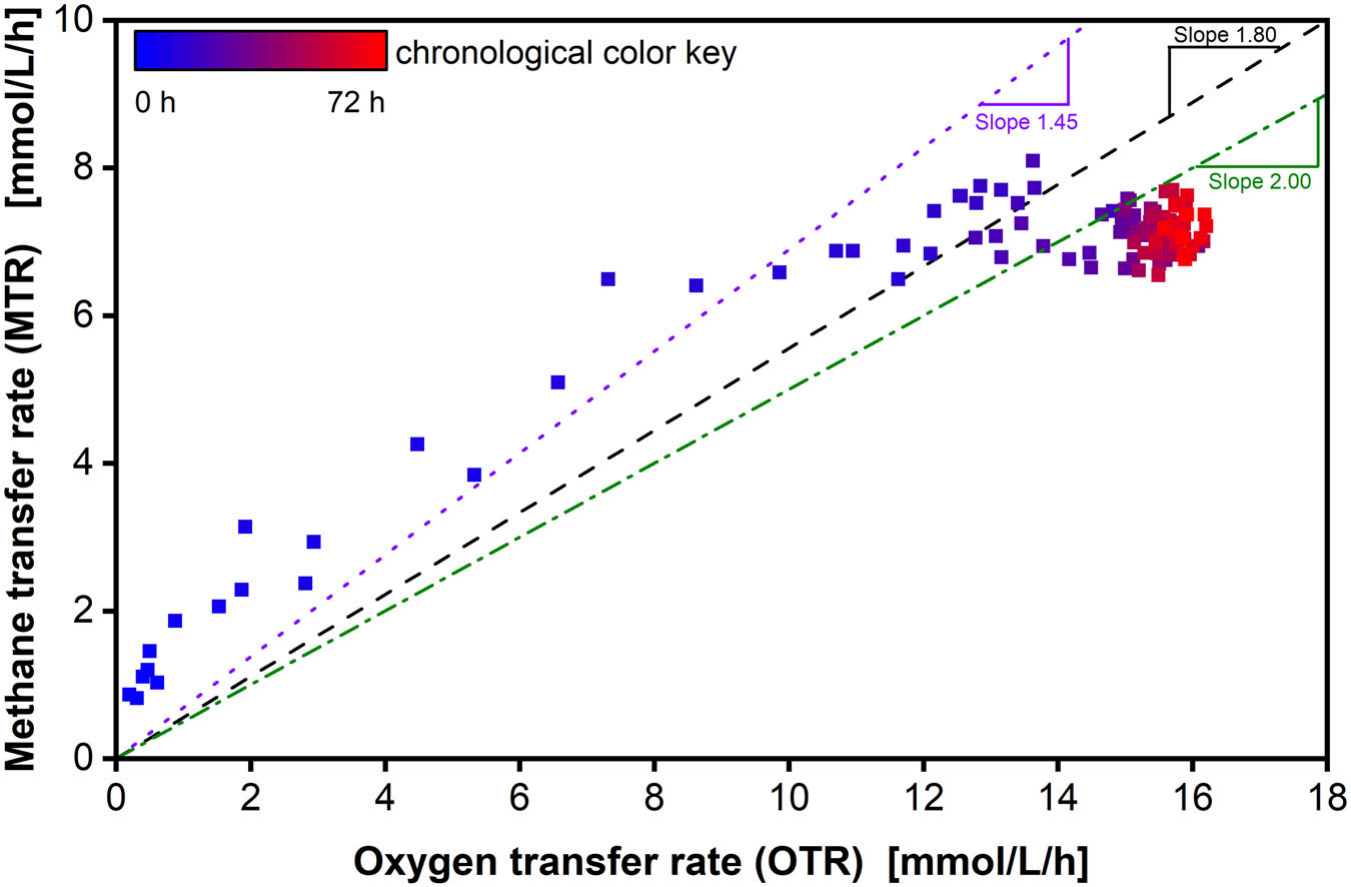
Oxidation quotient (OQ) of the cultivation over time of the cultivation in Figure 4. OQ in the form of the ratio of methane transfer rate (MTR) over oxygen transfer rate (OTR) during cultivation. The color shift from blue to red describes the temporal progression of cultivation time of the cultivation. The black dashed line indicates an $O_{2}$ to ${\text{CH}}_{4}$ ratio of 1.8. The red dotted line indicates an $O_{2}$ to ${\text{CH}}_{4}$ ratio of 1.45, which reflects the theoretical consumption rate during exponential growth rate (Petersen et al., 2019). The green dotted‐dashed line indicates an $O_{2}$ to ${\text{CH}}_{4}$ ratio of 2.0 for combustion.

For aerobic fermentations, the RQ is a well‐established quotient. It is defined as the ratio of the produced ${\text{CO}}_{2}$ and the consumed $O_{2}$ and gives detailed insights into the metabolism of cultures (García et al., 2020; Heyman et al., 2020; Müller et al., 2018; Romero‐Kutzner et al., 2015). Using different rates allows for the calculations of additional quotients with even further insights into product formation, such as the CORQ for carbon monoxide cultivations Mann et al. (2021). With the OTR and MTR of the methane RAMOS, the OQ and the CQ are calculable. The OQ describes the ratio of the consumed $O_{2}$ to ${\text{CH}}_{4}$ in the form of their respective transfer rates and should offer similar insights (see Figure 6). The initial values cluster slightly above an OQ of 1.45 for OTR values below 10 mmol/L/h during the exponential growth phase of the cultures. These findings are in line with the theoretical values for unlimited growth of 1.45 $O_{2}$ per ${\text{CH}}_{4}$, as described above. Existing models predict a shift in the ratios towards higher $O_{2}$ consumption during gas transfer limitations and agree with the monitored values as well (Lieven et al., 2018). At higher OTR values above 10 mmol/L/h, the OQ shifts towards 1.8, indicating a change in metabolism in the cultures. Comparing the values with Figure 4, it becomes obvious that the shift towards a ratio of 1.8 occurs during the gas transfer limitation phase indicated by the already described plateaus.

During that time, the cultures consume more $O_{2}$ per ${\text{CH}}_{4}$ and the transfer limitation restricts the oxidation of ${\text{CH}}_{4}$. The cultures switch away from biomass formation to a more energy‐efficient metabolism, which is reflected in the ratio of 1.8 $O_{2}$ per ${\text{CH}}_{4}$. Subsequently, a reduction in biomass formation is expected, as well. Since the organism would ideally consume the gases in the preferred ratio of 1.45, the fact that this ratio shifts towards a reduced ${\text{CH}}_{4}$ consumption per $O_{2}$ shows that $O_{2}$ is limiting. The available $O_{2}$ is increasingly used for energy conversion, instead for biomass formation. With further increasing biomass and a stable gas consumption, the $O_{2}$ per ${\text{CH}}_{4}$ ration increases further. Towards the end of the cultivation, a ratio slightly above 2.0 $O_{2}$ per ${\text{CH}}_{4}$ (see Equation 7) is reached. This shift suggests the halting or drastic reduction of biomass formation and a full switch towards maintenance metabolism. The CQ supports this as well, with values around 0.5 during the exponential growth phase (Figure S6). During the shift into the $O_{2}$ limitation, the CQ shifts towards a ratio of 0.7. During the limitation, the CQ increases to 0.9. This trend implies that *M. capsulatus* predominantly respires most of the assimilated carbon as ${\text{CO}}_{2}$, rather than utilizing it for biomass or product synthesis.

The online data generated by the methane RAMOS device enables the observation of metabolic shifts and the gas transfer limitations in shake flasks. This capability facilitates a detailed investigation into the metabolism of methanotrophic organisms and the influence of different parameters in small scale cultivations. The OQ introduces a new parameter for investigation of the metabolism of methanotrophic microorganisms. This additional metric provides valuable insights by tracking the metabolic shift from biomass formation to maintenance metabolism, thereby enhancing our understanding of metabolic behavior within this context.

### 3.4 Nitrogen fixation of *M. capsulatus*

**Figure 7:**
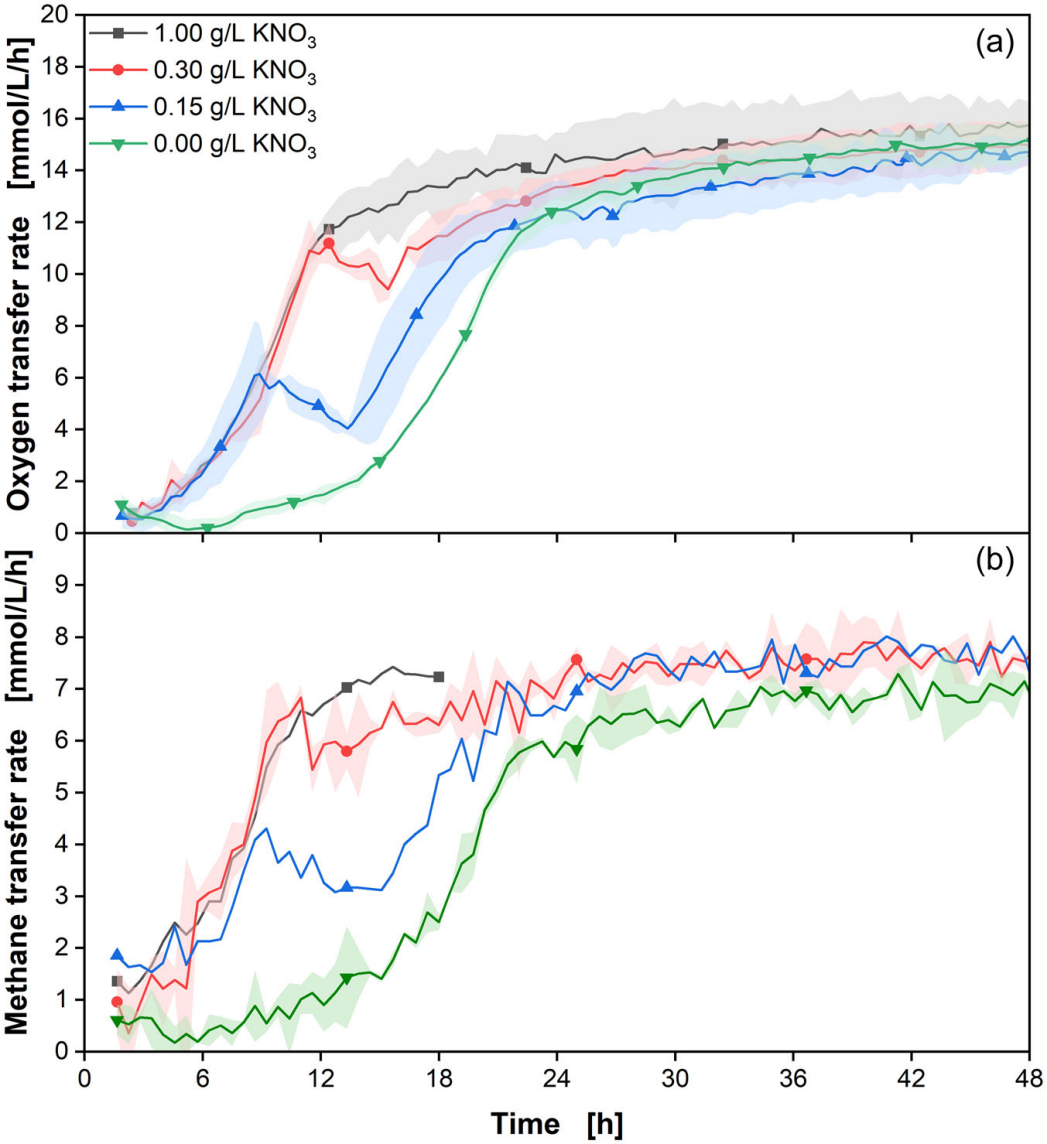
Comparison of transfer rates of *Methylococcus capsulatus* under various ${\text{KNO}}_{3}$ concentrations. (a) Oxygen transfer rate (OTR) and (b) methane transfer rate (MTR) courses of the first 48 h of *M. capsulatus* under various ${\text{KNO}}_{3}$ concentrations. The experiments were conducted in duplicates, with the average values represented as a line and the min/max values illustrated as error shadows. Due to sensor errors, the 1.0 and 0.15 g/L ${\text{KNO}}_{3}$ culture MTR data are only shown as singular courses. The MTR course of the 1 g/L ${\text{KNO}}_{3}$ culture is only shown for the first 18 h due to a faulty sensor. Before inoculation, washing the cells removed residual ${\text{KNO}}_{3}$ from the preculture. Cultivation conditions: NMS medium, $c_{N}$: 0–1 g/L ${\text{KNO}}_{3},c_{\text{buffer}}=30$ mM MOPS, $T=37^{\circ }C$, *n* = 350 rpm, $d_{0}=50$ mm, $V_{L}=10$ mL, initial OD = 0.1, initial pH = 6.8, *N* = 2. Only every 20th data point over time is indicated by the corresponding symbol; $c_{\text{in‐gas}}=5.9$ vol% ${\text{CH}}_{4}$, 3.4 vol% $O_{2}$, 1.3 vol% ${\text{CO}}_{2}$, 89.4 vol% $N_{2}$.

According to literature, *M. capsulatus* has the capability to fix inert $N_{2}$ from the atmosphere (Cui et al., 2022; Murrell & Dalton, 1983; Petersen et al., 2019; Zhivotchenko et al., 1995). To investigate the nitrogen fixation capabilities of *M. capsulatus* and further validate the methane RAMOS device, the nitrogen concentrations in the medium were varied from 0% to 100% of the default concentration of 1 g/L ${\text{KNO}}_{3}$. As a result, the medium contained ${\text{KNO}}_{3}$ concentrations of 0, 0.15, 0.3, and 1 g/L. The first 48 h of the OTR and MTR courses are shown in Figure 7. The whole courses are displayed in Figure S7.

Regardless of the supplied nitrogen concentration, all cultures exhibit growth, as indicated by increasing transfer rates. Nevertheless, significant differences in growth and lag phases among the different nitrogen concentrations are observable. Cultures with ${\text{KNO}}_{3}$ in the medium show a comparable exponential growth phase for the initial 9 h. After 9 h, the courses diverge depending on the supplied nitrogen concentration. Observable shifts occur in both the OTR and MTR courses. Following these shifts, the transfer rates continue to rise, resulting in an OTR plateau of around (15±0.99) mmol/L/h across all cultures. The shifts in the growth phases appear earlier with decreasing ${\text{KNO}}_{3}$ concentration, occurring at 9 h for 0.15 g/L ${\text{KNO}}_{3}$ and after 12 h for 0.3 g/L ${\text{KNO}}_{3}$, with OTR values around (5.9±0.9) and (11.1±0.49) mmol/L/h, respectively. These shifts in the transfer rates indicate the depletion of ${\text{KNO}}_{3}$ within the medium, representing the switch from nitrate consumption to nitrogen fixation from the atmosphere. The OTR and MTR integrals at the time of the shifts correlate with the provided nitrogen; doubling nitrogen concentrations results in a twofold increase in the corresponding OTR and MTR integrals (Figure S8). Calculating the total converted ${\text{CH}}_{4}$ until nitrogen depletion yields (17.8±0.7) mmol/L ${\text{CH}}_{4}$ for 0.15 g/L ${\text{KNO}}_{3}$ and (34.9±0.91) mmol/L ${\text{CH}}_{4}$ for 0.3 g/L ${\text{KNO}}_{3}$, corresponding to the theoretical nitrogen requirement of $116\;{\text{mmol}}_{{\text{CH}}_{4}}∕g_{{\text{KNO}}_{3}}$.

The 1 g/L ${\text{KNO}}_{3}$ cultures do not exhibit any shift. Theoretically, the shift should be visible after 26 h based on the ${\text{CH}}_{4}$ per ${\text{KNO}}_{3}$ fixation ratio. However, at this time, the plateau has already been reached, obscuring the shift. It is expected that the culture is nitrogen limited at this point as well, and that the cultures undergo nitrogen fixation from the atmosphere.

Cultures without nitrogen (0 g/L ${\text{KNO}}_{3}$) show a longer lag phase, with an exponential growth phase starting at around 6.5 h. These cultures reach an $O_{2}$ limitation at (15±0.8) mmol/L/h after 48 h. Both the cultures without ${\text{KNO}}_{3}$, as well as the cultures with reduced ${\text{KNO}}_{3}$ concentrations exhibit growth, showing an increase in the OTR signal following nitrogen depletion. The data demonstrates that *M. capsulatus* can grow without ${\text{KNO}}_{3}$ being present in the medium by fixing nitrogen from the atmosphere. Nevertheless, ${\text{KNO}}_{3}$ improves culture growth by increasing growth rates and significantly reducing the lag phase, due to the increased energy demand of the nitrogen fixation (Guo et al., 2022).

All cultures exhibit an $O_{2}$ limitation around the same OTR values of 15 mmol/L/h after 40–48 h, depending on the supplied concentrations of ${\text{KNO}}_{3}$. The courses are highly comparable to the experiments in the previous sections with similar OTR plateau values and growth rates.

After 100 h, the respiration of all cultures starts to cease and the OTR and MTR values decrease. This suggests that the cultures may be limited by a secondary substrate or by the accumulation of inhibitory metabolites, which have not been determined. Between 160 and 220 h, the OTR values start to decrease faster, nearing 0 mmol/L/h at the end of the cultivation. Once all OTR values reach around 1 mmol/L/h, the cultivations were stopped.

The MTR data consistently reflects the observed trends in the OTR data, further validating and providing insights into the experimental findings. Both the delayed growth for the 0 g/L culture, as well as the shifts in the growth phases for the 0.15 and 0.3 g/L cultures, are visible in the MTR data. The transfer rate ratios of the 1 g/L ${\text{KNO}}_{3}$ cultures are similar to the previous experiments, with an OQ shift from 1.45 towards 1.9 during the oxygen limitation (Figure S9). Cultures with reduced ${\text{KNO}}_{3}$ display an increased OQ to 1.9 significantly earlier during the nitrogen shift and maintain it until the end of the cultivation. The cultures without ${\text{KNO}}_{3}$ in the medium exhibit an OQ of 1.9 during the whole cultivation, indicating severe growth limitation and in turn reduced biomass formation. The assimilated carbon of all cultures, in the form of the CQ, also demonstrates this (Figure S10). The courses of the 1 g/L ${\text{KNO}}_{3}$ are highly comparable with the CQ courses from Section 3.2. During the unlimited growth phase, the CQ is around 0.6, indicating a balanced carbon metabolism, including biomass formation. Concurrently to the increasing OQ, the CQ increases to between 0.8 and 0.9, indicating a shift towards a metabolism prioritizing energy utilization over biomass formation. The nitrogen limited cultures show an CQ of around 0.9 from the beginning, indicating less carbon to biomass conversion, in addition to the prolonged lag phase. As reported, nitrogen fixation is an energy demanding process Jang et al. (2023), which explains the reduction in biomass formation and the increased CQ values. Online data monitoring using the methane RAMOS can effectively identify these changes, enabling the estimation of necessary nitrogen concentrations for optimal growth in a single shake flask experiment.

Cultures with ${\text{KNO}}_{3}$ in the medium exhibit an increased growth rate in the OTR values before reaching the ${\text{KNO}}_{3}$ depletion. Calculating the $\mu_{\max}$ using the formula from Stöckmann et al. (2003) results in a growth rate of $(0.37\pm 0.01)\;h^{-1}$ before nitrogen depletion and a reduced growth rate of $(0.23\pm 0.005)\;h^{-1}$ after the nitrogen depletion and for the 0 g/L ${\text{KNO}}_{3}$ cultures. These values align with the literature growth rates of nitrogen limited cultures ($0.25\;h^{-1}$) and unlimited cultures ($0.37\;h^{-1}$) (Joergensen & Degn, 1987). The reduced $\mu_{\max}$ of limited cultures suggests that nitrogen fixation is a limiting step in cell growth.

The end‐point pH values differ significantly among the different ${\text{KNO}}_{3}$ concentrations. Cultures with higher ${\text{KNO}}_{3}$ concentration show an increase in pH from the initial value of 6.8 to 7.05. In contrast, cultures with lower ${\text{KNO}}_{3}$ concentrations show a decreased pH, with lower values corresponding to lower ${\text{KNO}}_{3}$ concentrations (Table 1).

**Table 1:** End‐point pH of the cultivations of *Methylococcus capsulatus* under various ${\text{KNO}}_{3}$ concentrations.

	0.0 g/L ${\text{KNO}}_{3}$	0.15 g/L ${\text{KNO}}_{3}$	0.3 g/L ${\text{KNO}}_{3}$	1.0 g/L ${\text{KNO}}_{3}$
pH	6.48	6.67	6.77	7.05

*M. capsulatus* produces a variety of organic acids, such as acetate, lactate, succinate, and formate (Jang et al., 2023; Kalyuzhnaya et al., 2013). The production of organic acids increases significantly after cell growth ceased (Lee, 2021). Lower nitrogen concentrations in the cultures might cause reduced pH values due to premature limited cell growth, leading to increased organic acid production. HPLC measurements verified organic acids production below 1 g/L, namely acetate, for all cultures. The organic acid accumulation could also be the cause for the declining transfer rates at the end of the cultivation. An additional effect is the consumption of ${\text{KNO}}_{3}$ itself, which increases the pH upon metabolization, especially significant for the 1 g/L ${\text{KNO}}_{3}$ cultures. These two effects increase the final pH for higher ${\text{KNO}}_{3}$ concentrations and result in a lower pH for less ${\text{KNO}}_{3}$ and higher organic acid concentrations.

Overall, the data shows the capability of *M. capsulatus* to grow without nitrogen in the medium and the methane RAMOS can accurately monitor metabolic activities and limitations. With this system, the estimation of required nitrogen concentrations for optimal growth is possible, resulting in improved processes.

## 4 CONCLUSION

This study showcases the successful cultivation of methanotrophic organisms using the new methane RAMOS device. It enables the precise monitoring of all relevant transfer rates for methanotrophic cultivations, namely OTR, MTR, and CTR in up to eight shake flasks simultaneously. The insights generated by the transfer rates enhance the investigation of metabolic effects, limitations, and the influence of various cultivation parameters of methanotrophic organisms. The rapid and high‐resolution response of the ${\text{CH}}_{4}$ sensors ensures the accuracy of the MTR monitoring. Additionally, combining the closed gas balance and the GGTR evaluation with the monitored transfer rates improves the validation of the obtained rates.

The monitoring of transfer rates enables the online monitoring of the distinct stages of the process, including lag phases, growth, or depletions of secondary substrates. Throughout the cultivation processes, the RAMOS device can detect crucial gas transfer limitations as well. The observed $O_{2}$ transfer limitations and subsequent decreases in MTR indicate an increase in biomass and a heightened demand for maintenance energy by the cultures. Ratios like the OQ and the CQ provide additional insights into the metabolic state of the cultures. This setup also facilitates the detection of nitrogen limitations, as well as demonstrates nitrogen fixation of inert $N_{2}$ by *M. capsulatus*. A theoretical optimized nitrogen concentration in the medium could be estimated to improve biomass formation.

In summary, the setup described in this study not only enables the study of methanotrophic organisms but also streamlines the screening of various strains and experimental conditions. Its capacity to replicate conditions more akin to large‐scale fermentations with constant gas flow, in contrast to traditional serum flasks, accelerates the optimization of media and processes. Additionally, the device's online monitoring reduces manual labor, enabling in‐depth and precise analyses of up to eight parallel cultivations.

## AUTHOR CONTRIBUTIONS

**Dominik Engel**: Conceptualization (lead); methodology (lead); software (equal); investigation (lead); data curation (lead); writing—original draft (lead); visualization (lead). **Maximilian Hoffmann**: Investigation (supporting); data curation (supporting); writing—review and editing (supporting). **Udo Kosfeld**: Methodology (supporting); resources (lead); software (equal). **Marcel Mann**: Conceptualization (equal); supervision (lead); project administration (lead); writing—review and editing (equal).

## CONFLICT OF INTEREST STATEMENT

The authors declare no conflict of interest.

## Supporting information

Supplementary Information

## Data Availability

The data that support the findings of this study are available from the corresponding author upon reasonable request.
