# Supplementary material for: Online monitoring of methane transfer rates unveils nitrogen fixation dynamics in Methylococcus capsulatus
Source: Biotechnol Bioeng. 2024 Oct 11;122(1):110–22. doi: 10.1002/bit.28855 (PMC11632175; doi:10.1002/bit.28855)
Supplement: Supplementary file 1 — Supplementary Information [file BIT-122-110-s001.pdf]

Supplemental Materials

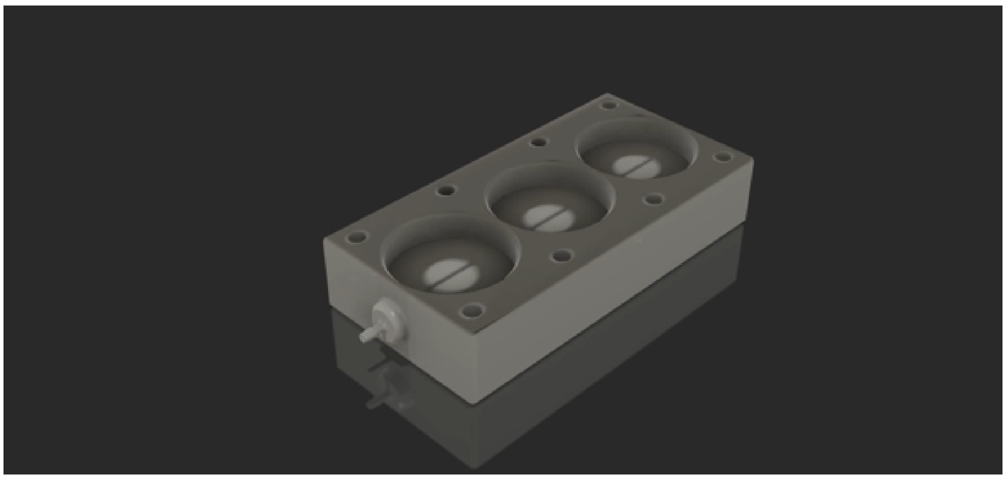

Figure S1: 3D printed sensor block.
The 3D printed sensor block used to house the sensors of the RAMOS device.

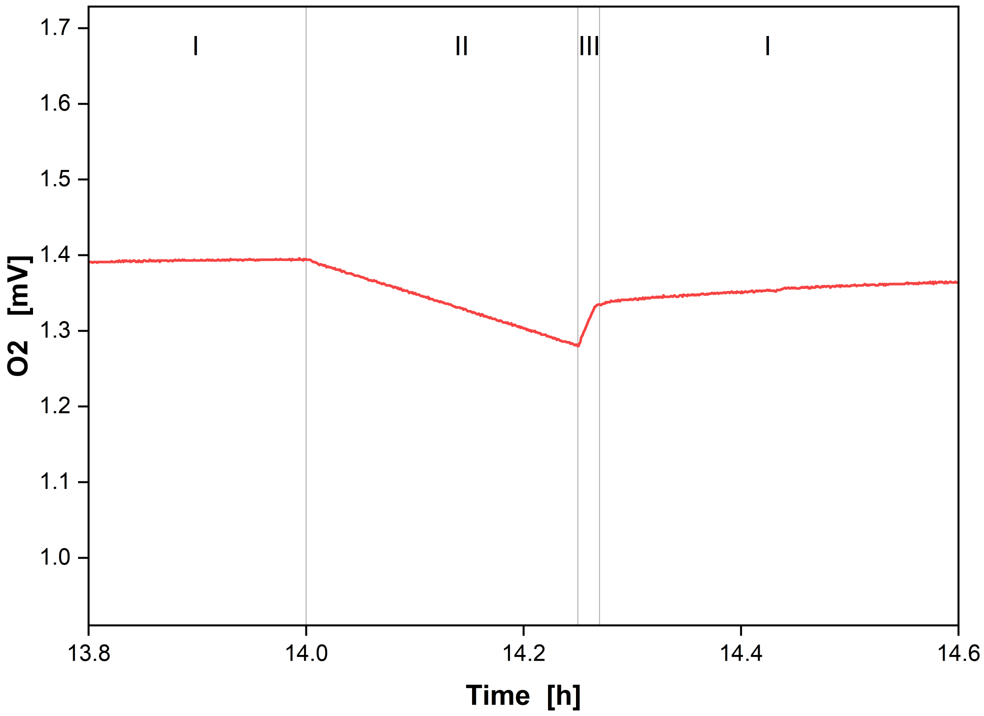

Figure S2: Raw oxygen sensor value during measurement phases.
I: Equilibration phase, II: Measurement phase, III: Flush phase. Progress of the oxygen partial pressure during a measurement phase. The O_2 content decreases up to 10 % during the measurement phase, resulting in a dynamically changing O_2 gradient.

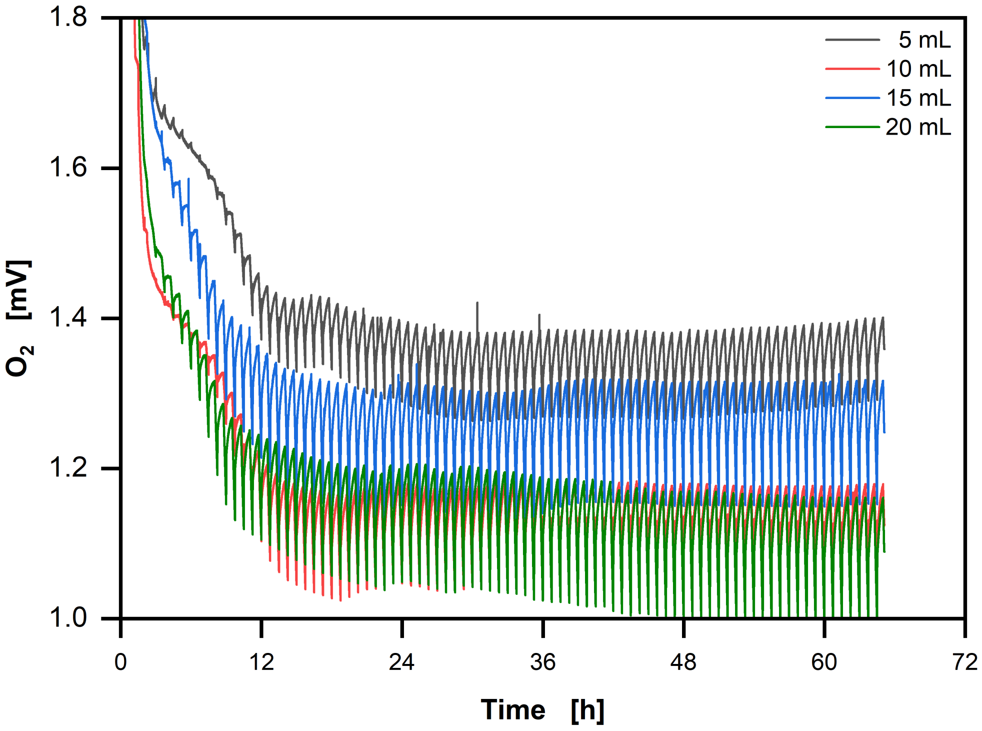

Figure S3: Raw oxygen sensor value of RAMOS experiment.
Progress of the oxygen partial pressure during the cultivation of Figure 3 with decreasing oxygen content for the first 12 to 14 hours.

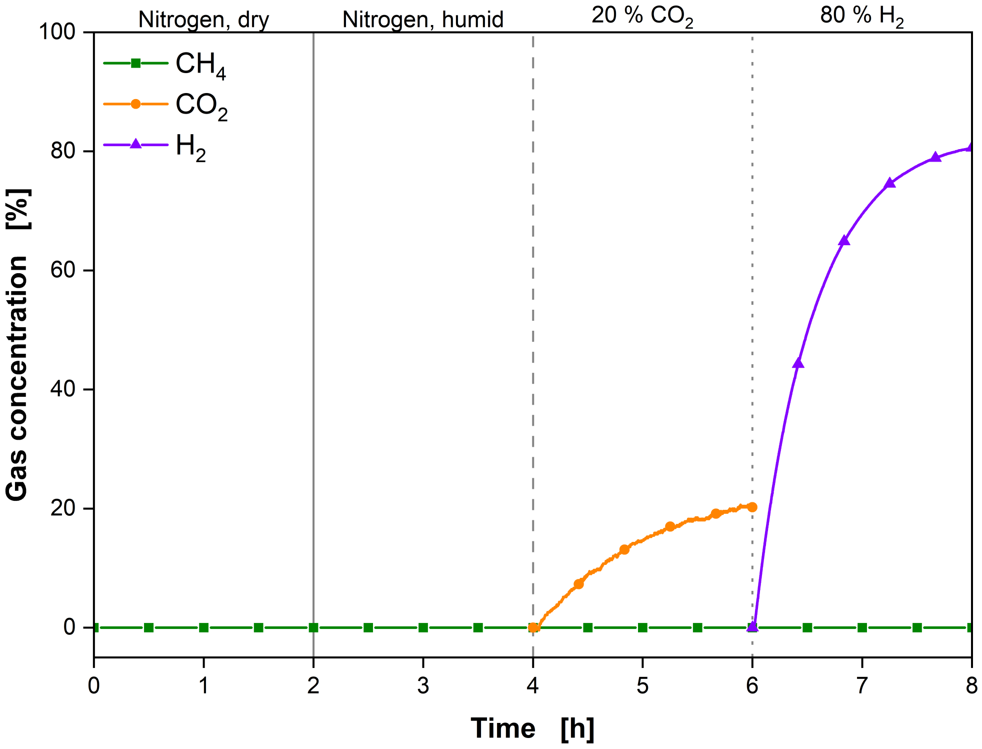

Figure S4: Effects of CO_2, H_2 and humidity on CH_4 sensor reading.
CH_4, CO_2, and H_2 sensor signals for different gas composition and humidity. 1. dry nitrogen gas; 2. humidified nitrogen gas; 3. CO_2 ramp from 0 – 20 vol%; 4. H_2 ramp from 0 – 80 vol%.

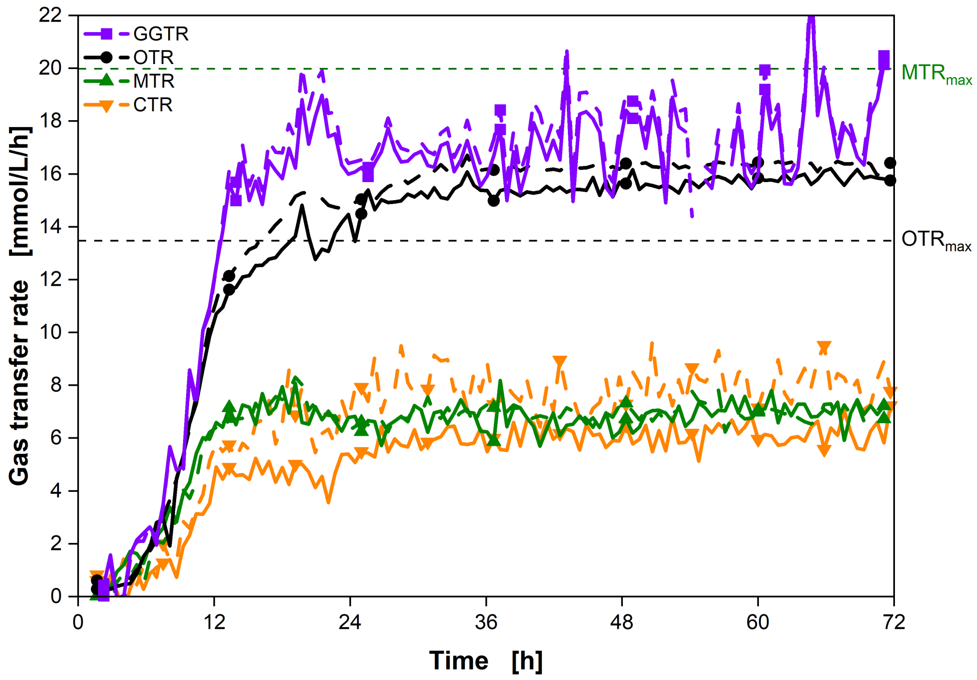

Figure S5: Single line data of Fig. 4.
Single line data of Fig. 4. Same color/icons symbolize same process parameters. Dashed lines indicate duplicates.

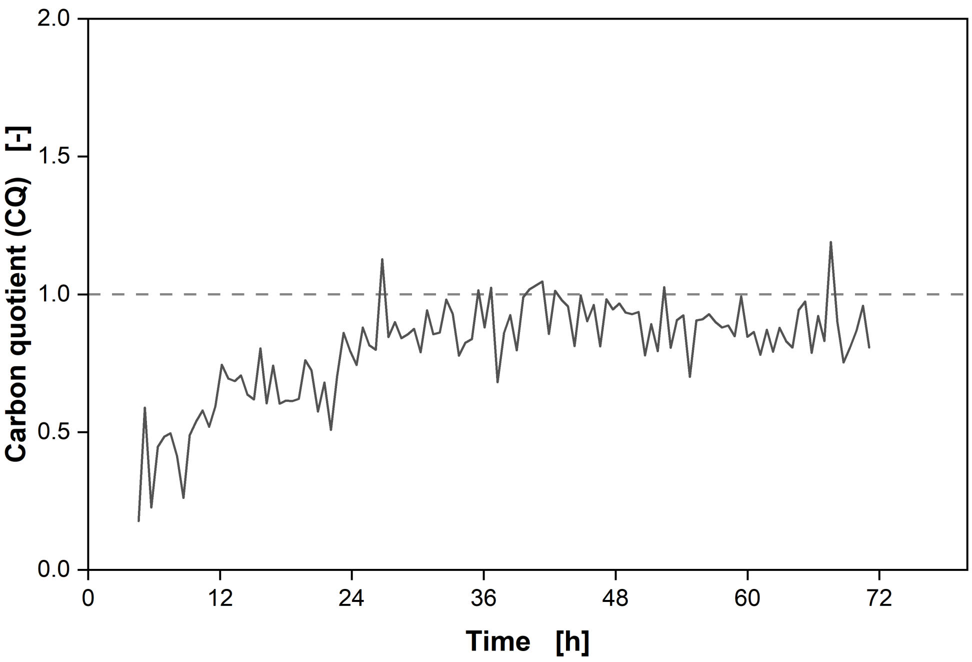

Figure S6: CQ of *M. capsulatus* cultivation.
CQ of the cultivation shifts from around 0.5 during the exponential growth phase towards 0.7 during the transition phase into the O_2 limitation. During the O_2 limitation, a CQ of around 0.9 can be measured.

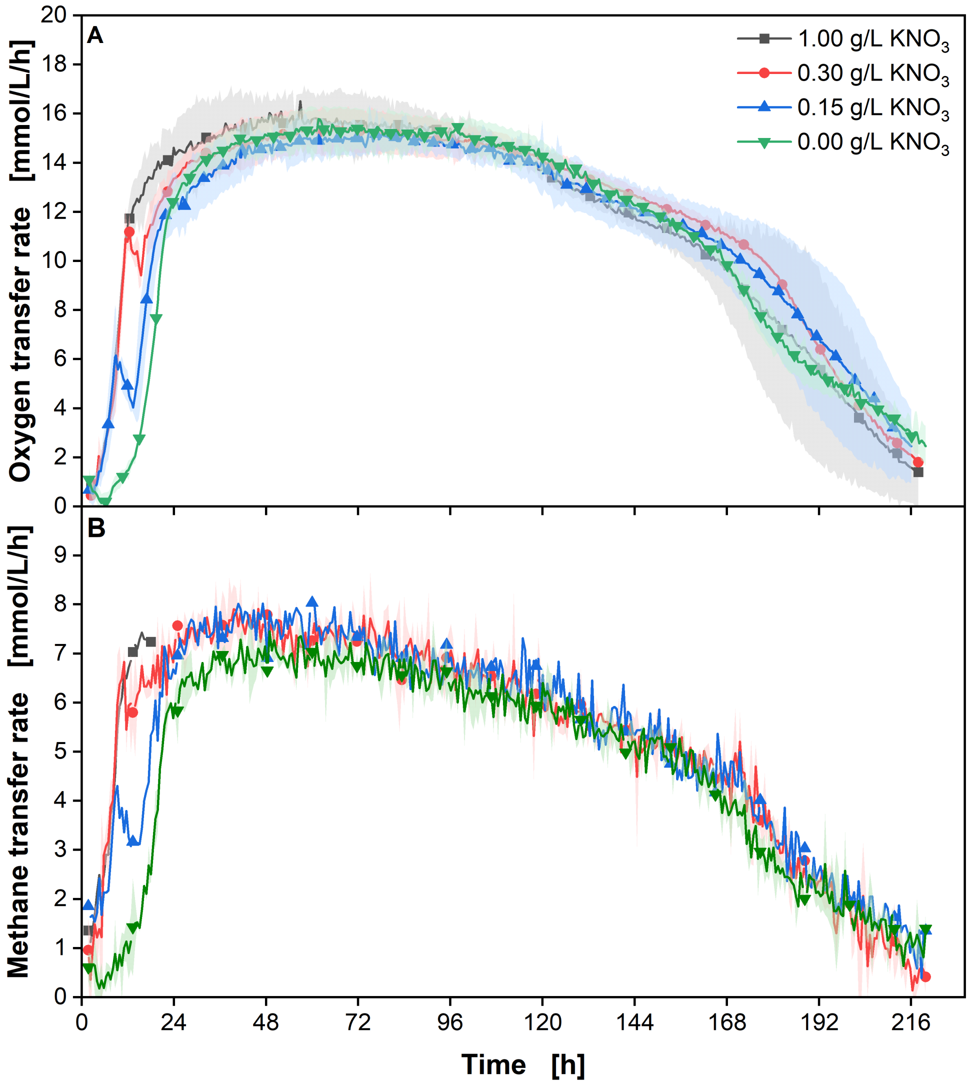

Figure S7: Comparison of transfer rates of *M. capsulatus* under various KNO_3 concentrations.

(a) OTR and (b) MTR courses of *M. capsulatus* under various KNO_3 concentrations. The cultivations were conducted in duplicates with the min/max values illustrated as shadows. Due to sensor errors, the 1.0 g/L and 0.15 g/L KNO_3 culture MTR data are only shown as singular courses. Before inoculation, washing the cells removed residual KNO_3 from the preculture. Cultivation conditions: NMS medium, c_N: 0 g/L – 1 g/L KNO_3, c_{buffer} = 30 mM MOPS, T = 37 °C, n = 350 rpm, d_0 = 50 mm, V_L = 10 mL, initial OD = 0.1, initial pH = 6.8, N = 2, Only every 20th data point over time is indicated by the corresponding symbol;
$c_{in\text{-}gas}$ = 5.9 vol% CH_4, 3.4 vol% O_2, 1.3 vol% CO_2, 89.4 vol% N_2.

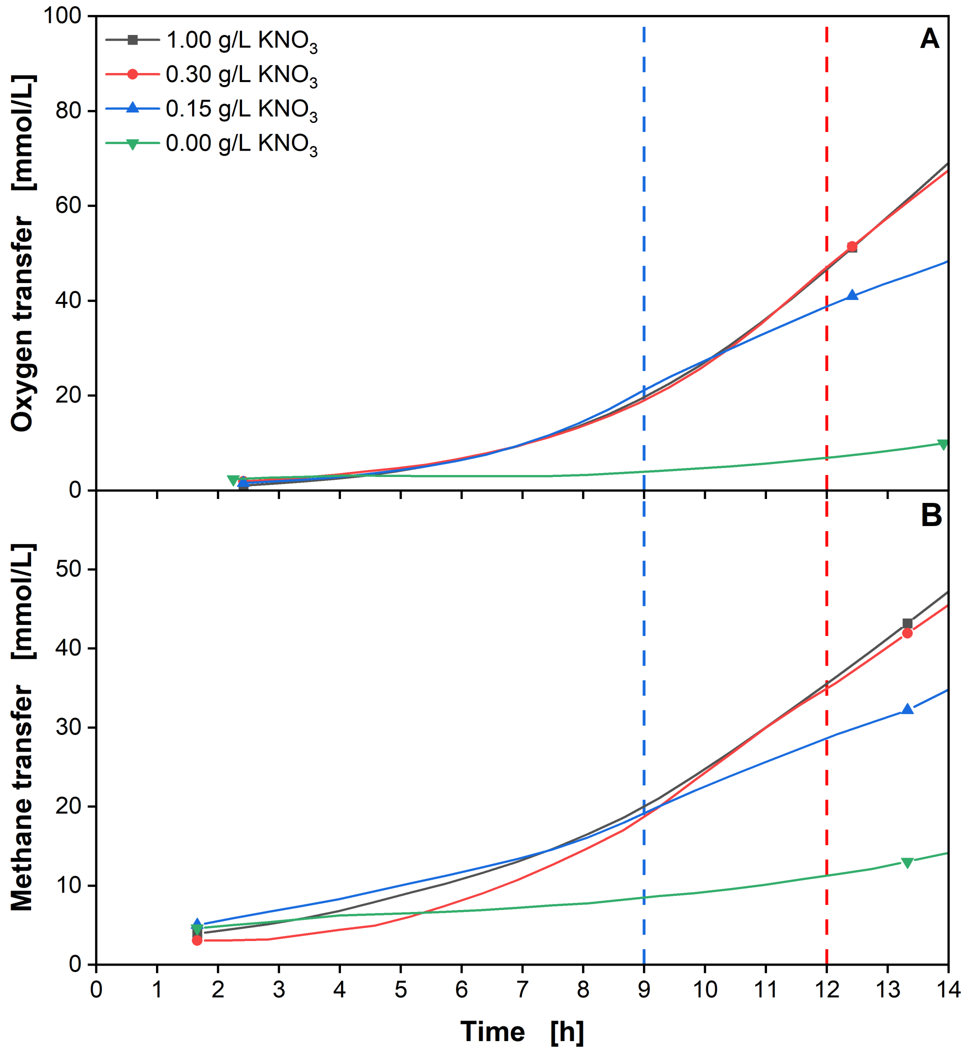

Figure S8: Integrals of transfer rates *M. capsulatus* under various KNO_3 concentrations.

Integrals of (a) OTR and (b) MTR courses of *M. capsulatus* under various KNO_3 concentrations. The time of the shifts for the 0.15 g/L KNO_3 and 0.3 g/L KNO_3 cultures are indicated by the vertical dashed lines. Cultivation conditions: NMS medium, c_N: 0 g/L – 1 g/L KNO_3, c_{buffer} = 30 mM MOPS, T = 37 °C, n = 350 rpm, d_0 = 50 mm, V_L = 10 mL, initial OD = 0.1, initial pH = 6.8, N = 2, Only every 20th data point over time is indicated by the corresponding symbol;

$c_{in\text{-}gas}$ = 5.9 vol% CH_4, 3.4 vol% O_2, 1.3 vol% CO_2, 89.4 vol% N_2.

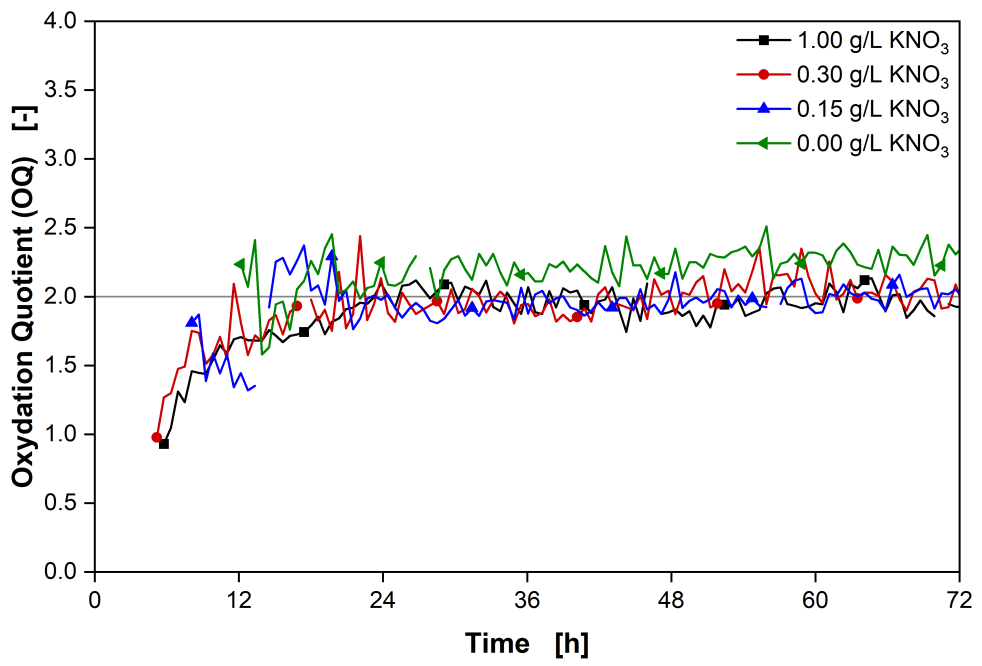

Figure S9: OQ of *M. capsulatus* cultivation under various KNO_3 concentrations. OQs of the cultivation first 72 hours of the cultivation are shown. Both 1 g/L and 0.3 g/L KNO_3 start with an OQ of 1.0 and settle around 1.5 before the oxygen limitation. Cultures with 1 g/L KNO_3 reaches the OQ of 1.9 after 24 hours, while 0.3 g/L KNO_3 reaches the same OQ after 15 hours shortly after the nitrogen shift. The cultures with 0.15 g/L KNO_3 start around 1.45 and reach 1.9 after 13.5 hours. If no KNO_3 is present in the medium, the OQ starts at 1.9 from the beginning once the calculation threshold of OTR > 2 mmol/L/h is reached. Ratios with OTR values above 2.0 are shown. Only every 20th data point over time is indicated by the corresponding symbol.

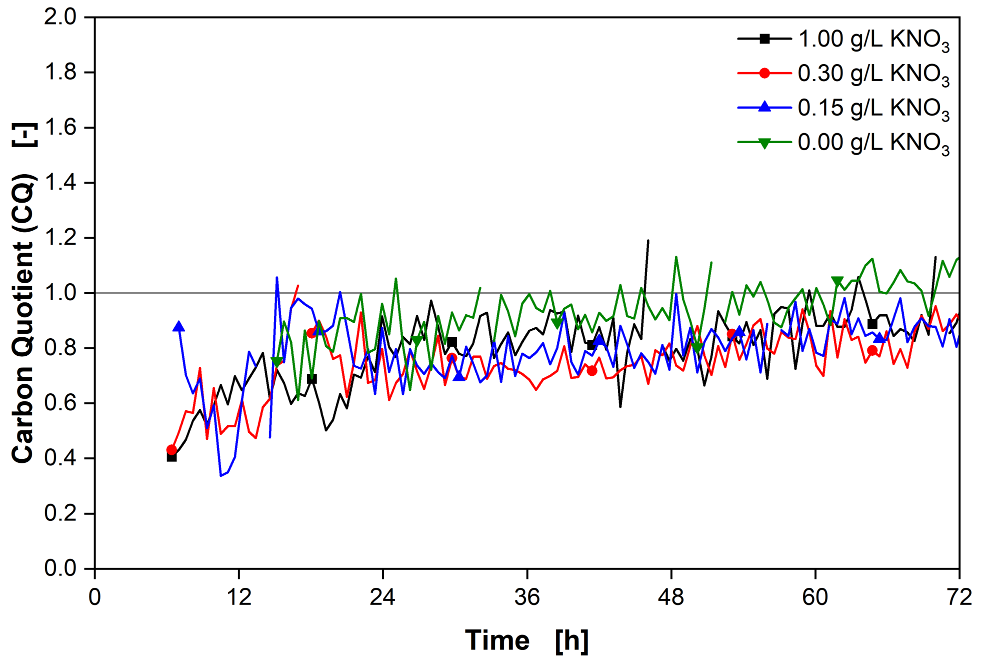

Figure S10: CQ of *M. capsulatus* cultivation under various KNO_3 concentrations. CQs of the cultivation first 72 hours of the cultivation are shown. Both 1 g/L and 0.3 g/L KNO_3 start with a CQ of 0.5 to 0.6 and settle around 0.7 to 0.8 after the oxygen limitation. Cultures with 1 g/L KNO_3 reaches the CQ of 0.9 after 24 hours, while 0.3 g/L KNO_3 reaches the same CQ after 15 hours shortly after the nitrogen shift. The cultures with 0.15 g/L KNO_3 start around 0.6 and reach 0.8 after 13.5 hours. If no KNO_3 is present in the medium, the CQ starts at 0.8 from the beginning once the calculation threshold of CTR > 1 mmol/L/h is reached. Ratios with CTR values above 1.0 are shown. Only every 20th data point over time is indicated by the corresponding symbol.
